# Spatio-temporal evolution law of gas-temperature coupling field in “110 method” goaf and prevention of spontaneous combustion

**DOI:** 10.1371/journal.pone.0293829

**Published:** 2023-11-20

**Authors:** Song Wei, Zhenqing Fang, Zongxiang Li, Yu Liu, Dongjie Hu, Chuntong Miao, Haiwen Wang

**Affiliations:** 1 Liaoning University of Technology, Liaoning, China; 2 College of Safety Science and Engineering, Liaoning Technical University, Liaoning, China; 3 Key Laboratory of Mine Thermodynamic disasters and Control of Ministry of Education, Liaoning Technical University, Liaoning, China; 4 China Coal Technology and Engineering Group Shenyang Research Institute, Fushun, China; 5 Ansteel Group Mining Co. LTD, Liaoning, China; Kanazawa University: Kanazawa Daigaku, JAPAN

## Abstract

To investigate the distribution characteristics of spontaneous combustion disaster (SCD) zones in the goaf of "110" mining method with U + L ventilation system and formulate corresponding fire prevention measures, enclosed coal oxidation experiments were carried out to measure the oxidation characteristics of Dongrong Coal Mine bituminous coal sample. A coupled 3DEC-CFD (3 dimensional Distinct Element Code) model was established. The 3D transient distribution characteristics of SCD zones in the “110” mining goaf under U+L ventilation condition were analyzed. Nitrogen injection in the tailgate was proposed for coal spontaneous combustion prevention. The results show that air leakage changed the distribution of oxygen and temperature fields in the “110” goaf, causing the region 20~60 m parallel to the retained roadway to remain in the oxidation zone for spontaneous combustion. As the working face advanced, the area change curve of SCD zones in the “110” goaf exhibited a “double inflection point” pattern. Eliminating the “retained roadway oxidation zone” can effectively reduce the spontaneous combustion risks in the “110” goaf and ensure mining safety. This study enriches the mechanisms of coal spontaneous combustion.

## 1. Introduction

The “110 method,” which is typically called the roof-cutting gob-side entry retaining (RCGSER) technology, is a third-wave technological advancement in China’s mining industry [1, 2]. However, the technology requires a direct connection of the retained roadway and goaf, and the air leakage range and rate increase with increasing goaf strike length [3, 4]. Hence, the distribution of the air flow field and temperature field in the goaf also change in real time. Understanding the dynamic distribution characteristics of spontaneous combustion zone in the goaf of RCGSER is of great significance to prevent and control spontaneous combustion and ensure the safety of coal mine production.

Currently, the steady-state numerical simulation method is used in studying the distribution of spontaneous combustion danger (SCD) areas in goafs (The area with the possibility of spontaneous combustion in the goaf) [5–10]. The method assumes that the space and porosity distribution of the goaf do not change with increasing goaf strike length. Hongwei [11] obtained the distribution characteristics of the spontaneous combustion zone of goafs under U + L and W ventilation in the RCGSER working face through simulation analysis. The author proposed a fire prevention and extinguishing technology of spray plugging in the retained roadway. Guoqing [12] proposed an unsteady simulation method of a “fixed grid and dynamic attribute” for a dynamic mining process from a four-dimensional perspective of space and time, and the author identified two stages in which the distribution range of the “oxidation zone” changes with increasing goaf strike length. Yanqing [13] established a calculation model of gas seepage of residual coal in the goaf based on moving coordinates; the model accurately represented the gas emission of the working face through simulation. Yansong [14] used the discrete element modeling software PFC to simulate the evolution and pore distribution of the overlying strata of the goaf after mining; a dynamic porosity model of the goaf was developed, and the airflow distribution in the goaf. In the above research, the boundary of the simulation model has not changed. However, the open RCGSER goaf formed after roof cutting is directly connected to the retained roadway, and the air leakage range from the retained roadway to the goaf varies with increasing goaf strike length. Therefore, changing the model boundary with time in the numerical simulation is more consistent with the real working face advancement. Temperature in the goaf is vital for determining the danger of spontaneous combustion in the goaf [14–20]. The heat released from the reaction between residual coal and oxygen in the goaf increase the coal temperature; thus, the temperature is related to the oxygen consumption rate and the oxygen consumption of residual coal. Because the oxygen content in the air varies across the goaf, it is necessary to determine the oxygen consumption rate of residual coal under different oxygen concentrations. But the above studies rarely involve the changes of airflow and temperature fields in the cut-through along-retained roadway goaf over time, and most of them use empirical values (5%, 8%, 10%) to determine the critical oxygen concentration for goaf inertisation, which should be different for coals of varying qualities.

Therefore, the S2173 RCGSER first working face in the 17# coal seam (Bituminous coal) of Dongrong Mine was investigated in this study. A thermogravimetric analysis, temperature programmed test, and closed oxygen consumption test were used to measure the thermogravimetric characteristics of coal samples, temperature of each landmark gas, oxygen consumption rate under different oxygen concentrations, and shortest spontaneous combustion period. The measurements were combined with the simulation results of the 3DEC (3 Dimension Distinct Element Code) discrete element software to establish a four-dimensional porosity model (UDF) of the goaf. The dynamic and sliding grids in the computational fluid dynamics (CFD) software were used to achieve a moving boundary of the simulation model at a certain speed, simulating the advancing of the working face. Thus, we analyzed the spatio-temporal changes in the distribution characteristics of the gas flow field and temperature field in the goaf as the working face advances. Consequently, we proposed the measure of injecting nitrogen into the tail roadway to prevent spontaneous combustion in the goaf.

## 2. Experiment and methods

### 2.1 Thermogravimetric analysis experiment

A synchronous thermal analyzer (STA6000, PerkinElmer) was used to study the thermogravimetric process of coal samples during oxidation. In the experiment, 10–20 mg of coal samples were weighed and put into the crucible; the air flow rate was 40 ml/min, the temperature range was 30–700°C, and the heating rate was 10°C/min.

### 2.2 Temperature programmed experiment

**Fig 1:**
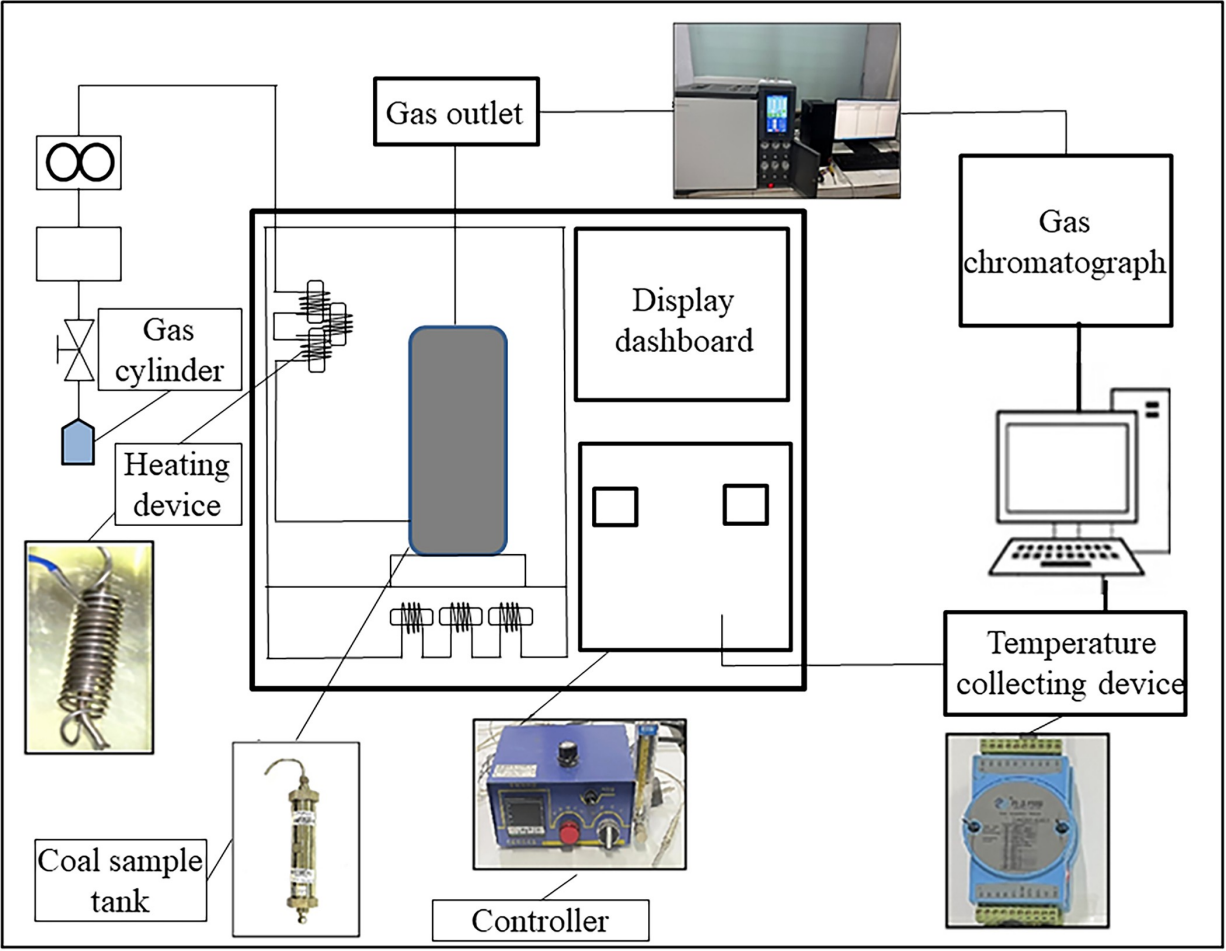
Schematic of temperature programmed experimental device.

A self-made heating device (Fig 1) was used to conduct the temperature programmed test. The device was composed of a temperature controller, data acquisition board, air pump, cooling pipe, temperature compensator, and other components. The temperature control accuracy was less than 0.5°C, and it was used in combination with a gas chromatograph. During the experiment, an 80-g coal sample was canned, and the temperature range was set at 30–500°C (the experiment was stopped when acetylene gas appeared).

### 2.3 Closed oxygen consumption test

**Fig 2:**
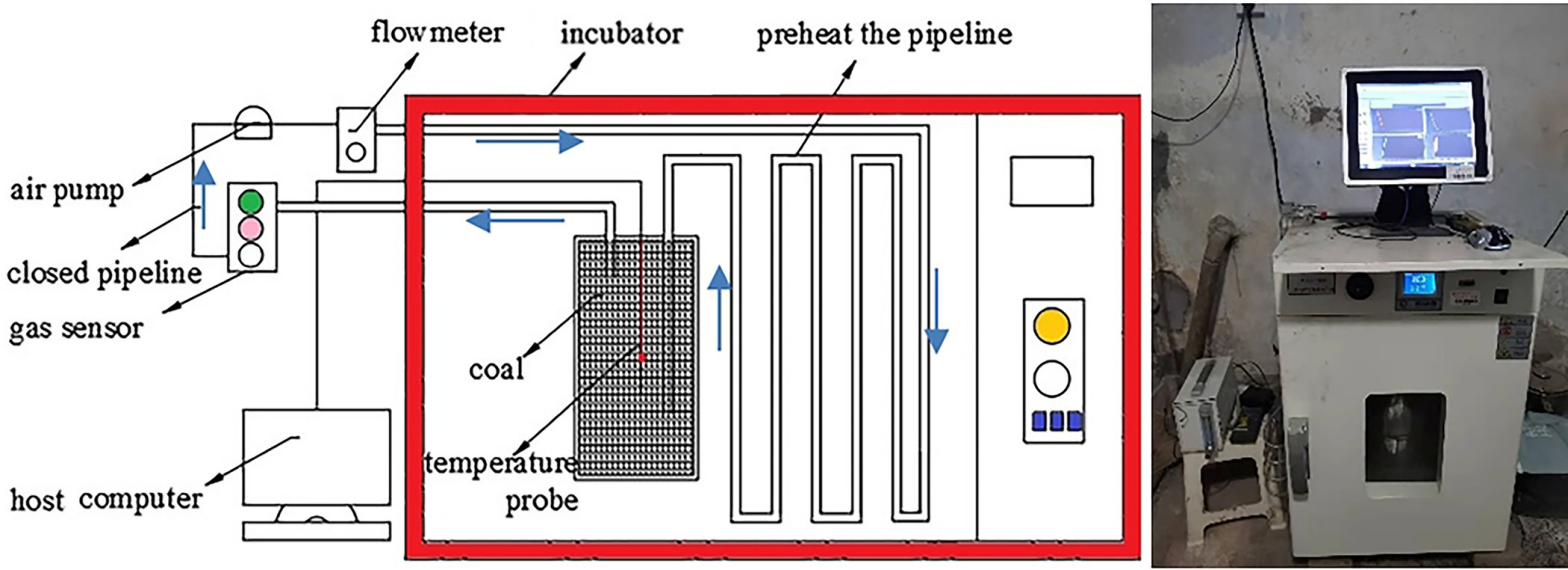
Schematic of closed oxygen consumption experimental device.

The test device is shown in Fig 2; coal samples from Jixiang Mine and Hongqingliang Mine are added as control group. The coal samples were prepared by pulverizing large coal samples stripped off the surface oxide layer; coal samples with a particle size ranging between 0.425 mm and 2.00 mm were sieved out and sealed in vacuum for later use. Finally, 2,000 g of the coal samples was used for the experiment.

When measuring the continuous oxygen consumption rate of the coal samples, the temperature of the incubator was set to the underground ambient temperature of Dongrong Mine (298 K). Disconnect the pipeline for 10 minutes before the test to purge interferences. After purging is completed, connect the test pipeline, check the test device to ensure no air leaks in the pipeline, and run the constant temperature box to keep its temperature consistent with the average temperature of the working face. Open the data acquisition software to record O_2_ and CO concentration data. Stop the experiment when the change of the data curve tends to be stable.

When measuring the spontaneous combustion period of the coal samples, the closed pipeline was opened, and the closed oxygen consumption experiment was changed into the temperature rise experiment by passing air into the test device. The thermostat was closed while the temperature change of the coal samples during natural oxidation was recorded in real time. Finally, the spontaneous combustion period was determined according to the experimental results of the programmed temperature rise.

### 2.4 Measured field data

**Fig 3:**
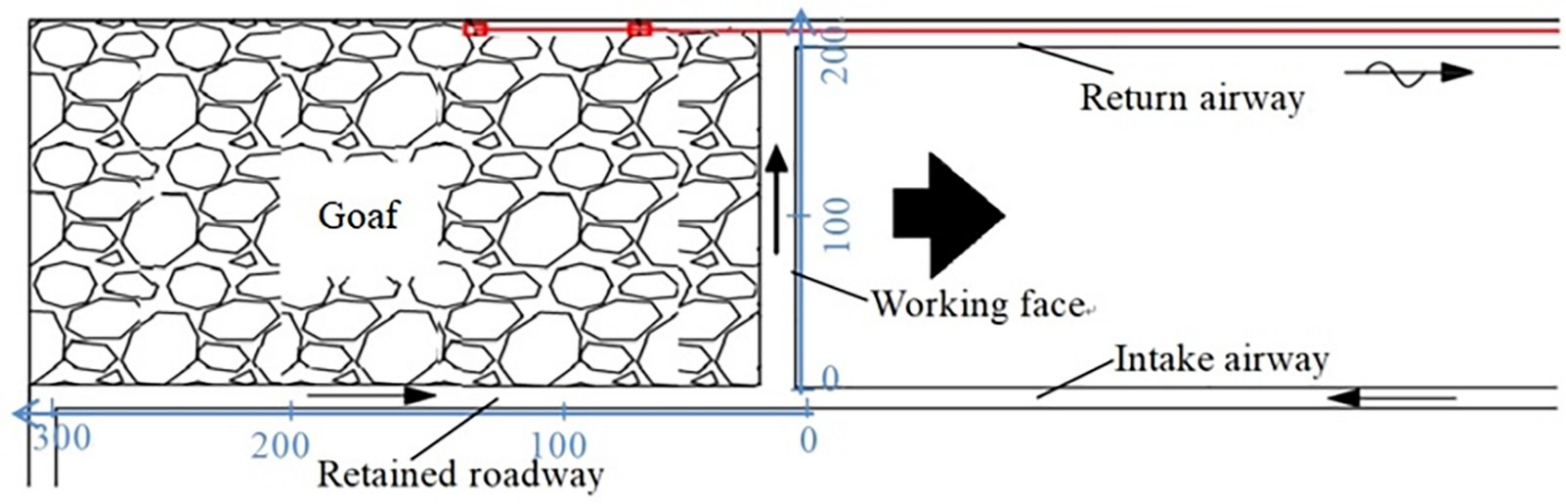
Layout of working face and burial form of bundle pipe.

The dip length of the working face was 200 m, and an air volume measuring point was set at every 3 m. The length of the retained roadway was 300 m, and an air volume measuring point was set at every 7 m. The air volume data of more than 110 measuring points were obtained. Bundle tubes and AD590 temperature sensors were embedded in the return-air lane to measure the change in gas composition and temperature in the goaf. Dongrong Mine is a low-gas mine, and the working face layout and bundle pipe embedding mode are illustrated in Fig 3.

### 2.5 Simulation method

#### 3DEC discrete element simulation software

The 3DEC simulation software was combined with the main physical and mechanical properties of rock (Table 1) to simulate and analyze the caving and deformation of overlying strata in the S2173 RCGSER working face of Dongrong Mine during excavation at 60, 120, 240, 300, and 420 m. The dip angle of the 17# coal seam in Dongrong Mine was 18, the caving step was 20 m, the width of the boundary coal pillar was greater than 100 m, and the overlying strata were 200 m thick (a load of 400 m strata is applied).

**Table 1:** Main physical and mechanical properties of rock.

Rock name	Bulk density kg/cm^3^	Porosity %	Compressive strength 102 kg/cm^3^	Tensile strength 102 kg/cm^3^	Deformation modulus 102 kg/cm^3^	Elastic modulus kg/cm^3^	Poisson’s ratio
Sandstone	2.0–2.60	5–25.00	2–20.00	0.50–0.40	0.50–8.00	1.00–10.00	0.28
Conglomerate	2.3–2.60	5–15.00	1–15.00	0.20–1.50	0.80–8.00	2.00–8.00	0.30
Mudstone	2.7–2.85	1.6–5.20	1–12.00	0.60–2.00	2.00–7.00	5.00–10.00	0.23
Limestone	2.2–2.70	5–20.00	5–20.00	0.50–2.00	1.00–8.00	5.00–10.00	0.29
Shale	2.0–2.40	16–30.00	1–10.00	0.20–1.00	1.00–3.50	2.00–8.00	0.30
Quartz	2.65–2.70	0.12–0.50	15–35.00	1.0–3.00	6.00–20.00	6.00–20.00	0.32

#### CFD simulation

**Fig 4:**
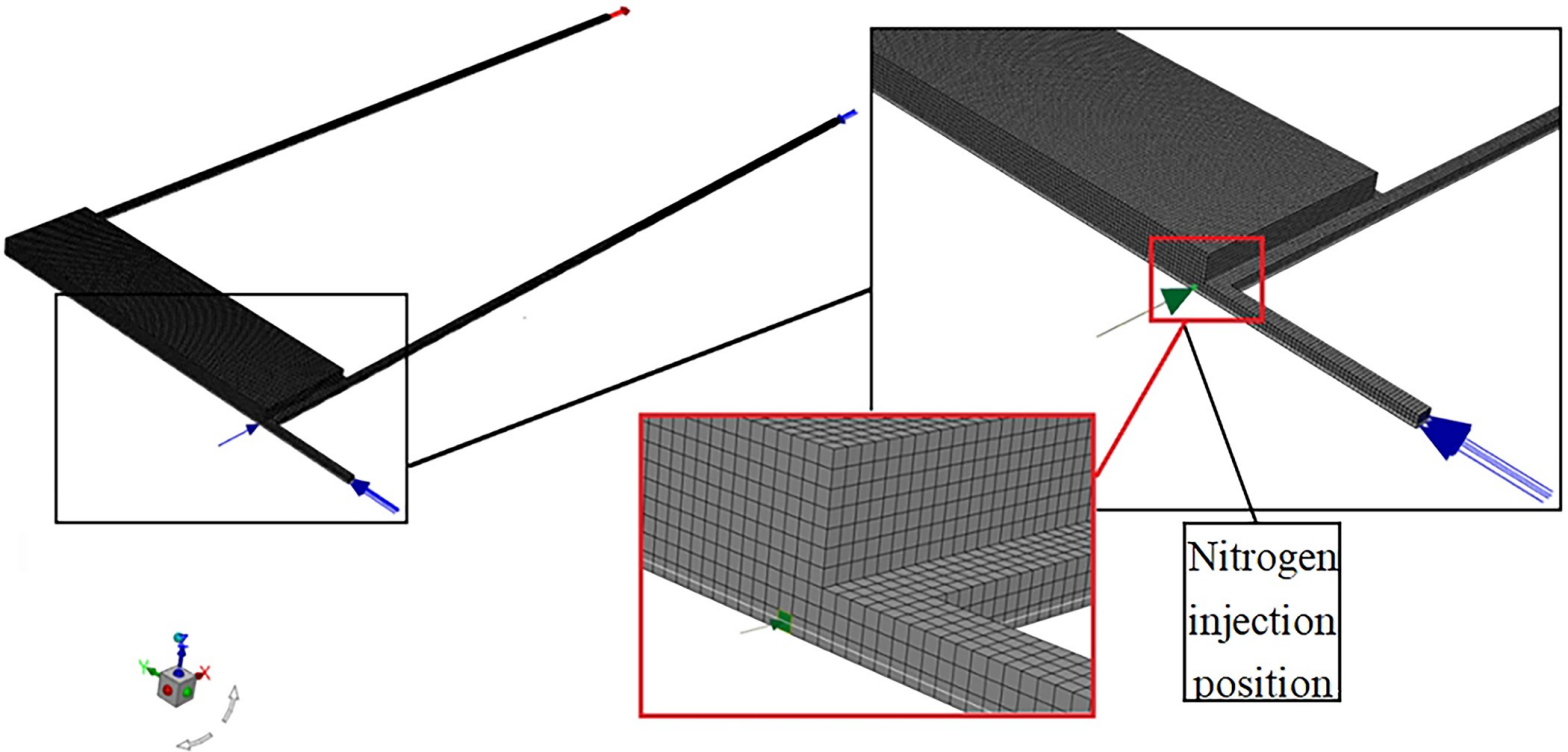
Initial mesh model.

Fig 4 shows the initial simulation grid model: the nitrogen injection point is near the tail roadway, with a roadway width of 4 m, height of 3 m, and goaf height of 20 m. The dip angle of the coal seam was realized by changing the direction of gravitational acceleration during simulation, and the height of the oxygen consumption layer of residual coal was set to 0.5 m. In CFD, sliding grid technology and dynamic layer technology were used simultaneously: the velocity (0.00005 m/s) and direction were controlled by UDF, the collapse factor was 0.2, the splitting factor was 0.4, the time step was 10,000 s, and the dynamic advancing process of the working face from 60 m to 600 m was simulated.

## 3. Results and analysis

### 3.1 Experimental results of thermogravimetric analysis

**Fig 5:**
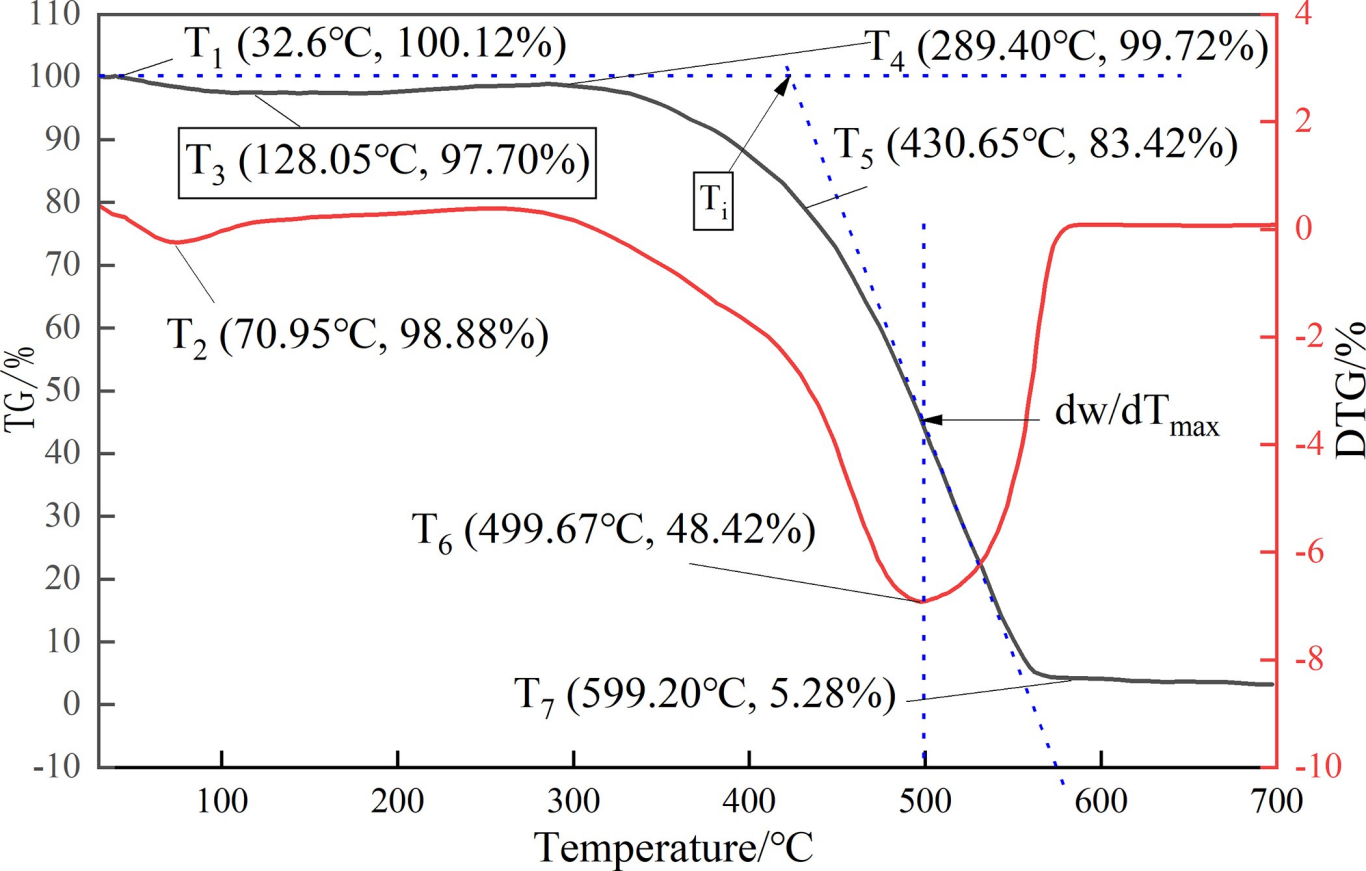
TG and DTG curves of coal samples.

Fig 5 shows the characteristic temperature and corresponding mass fraction change curve of coal samples in Dongrong Mine. According to the characteristic temperature, the heating and oxidation process of coal samples in Dongrong Mine can be divided into four stages: preheating weight loss stage (30°C–T_3_), oxidation weight gain stage (T_3_–T_4_), combustion stage (T_4_–T_6_) and burnout stage (T_6_−700°C).

**Fig 6:**
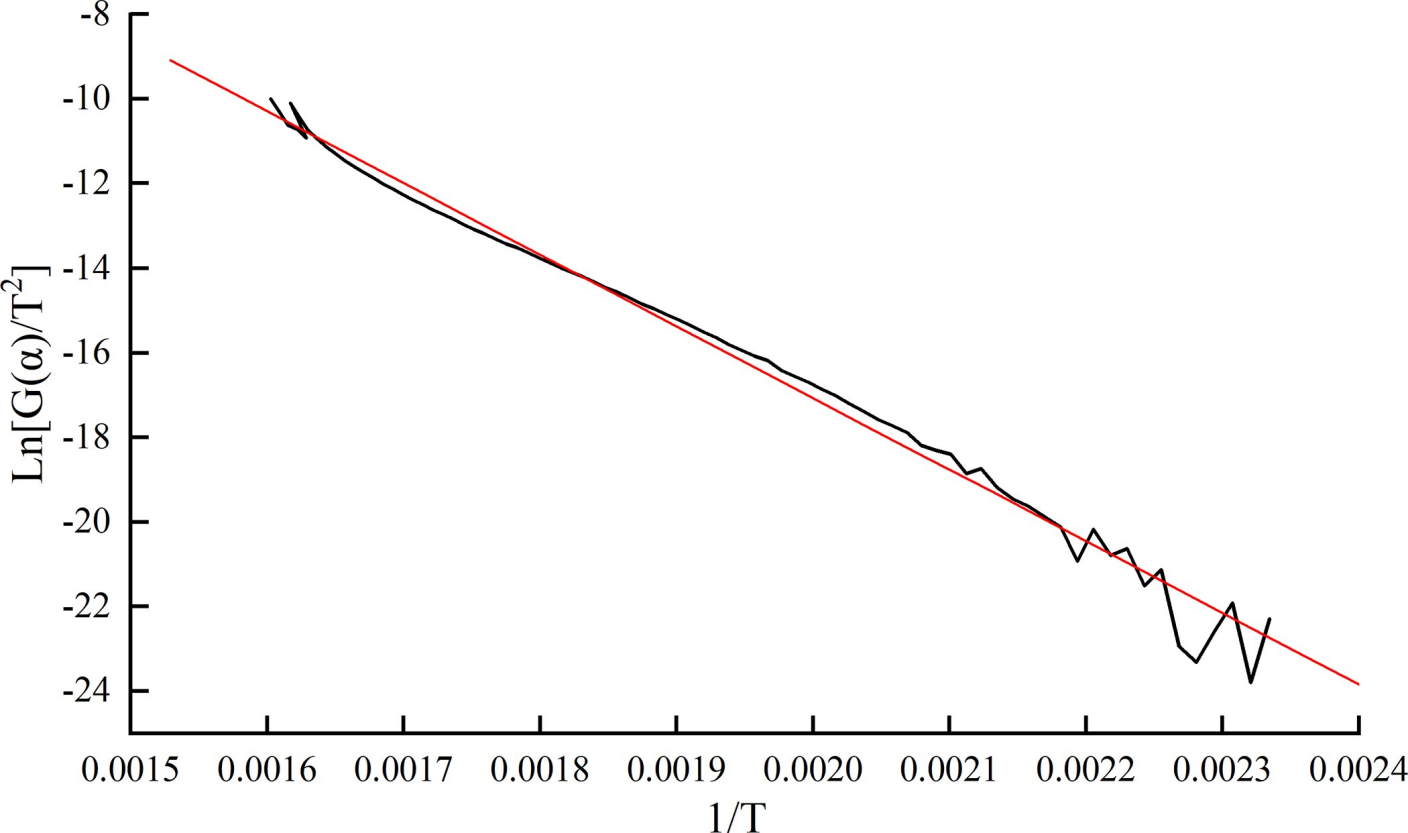
Relationship between ln[G(α)/T^2^] and 1/T.

The endpoint temperature of water loss and ignition temperature of the 17# coal in Dongrong Mine were approximately 170°C and 320°C respectively. The relationship between ln[*G*(*α*)/*T*^2^] and 1/*T* was obtained from the thermogravimetric experiment of the 17# coal sample, as shown in Fig 6.

The dynamic parameter equation of the spontaneous combustion ignition stage is as follows:

Y=16.97792−16929.99674X, (1)

where the correlation of linear regression is 0.99517; the activation energy E = 140.7 kJ/mol (T_3_-T_4_).

Basic equations of thermal analysis kinetics [21]:

$$\int_{0}^{\alpha }\frac{d\alpha }{f(\alpha )}=G(\alpha )=\frac{A}{\beta }\int_{T_{0}}^{T}\exp(-\frac{E}{RT})dT,$$ (2)

where *α* is the conversion percentage, dimensionless; *f*(*α*) and *G*(*α*) are differential and integral dynamic mechanism functions; *T* is the reaction temperature, K; *T*_0_ is the starting temperature, K; *E* is the reaction activation energy, kJ/mol; and *R* is the molar gas constant.

### 3.2 Results of temperature programmed experiment and analysis

**Fig 7:**
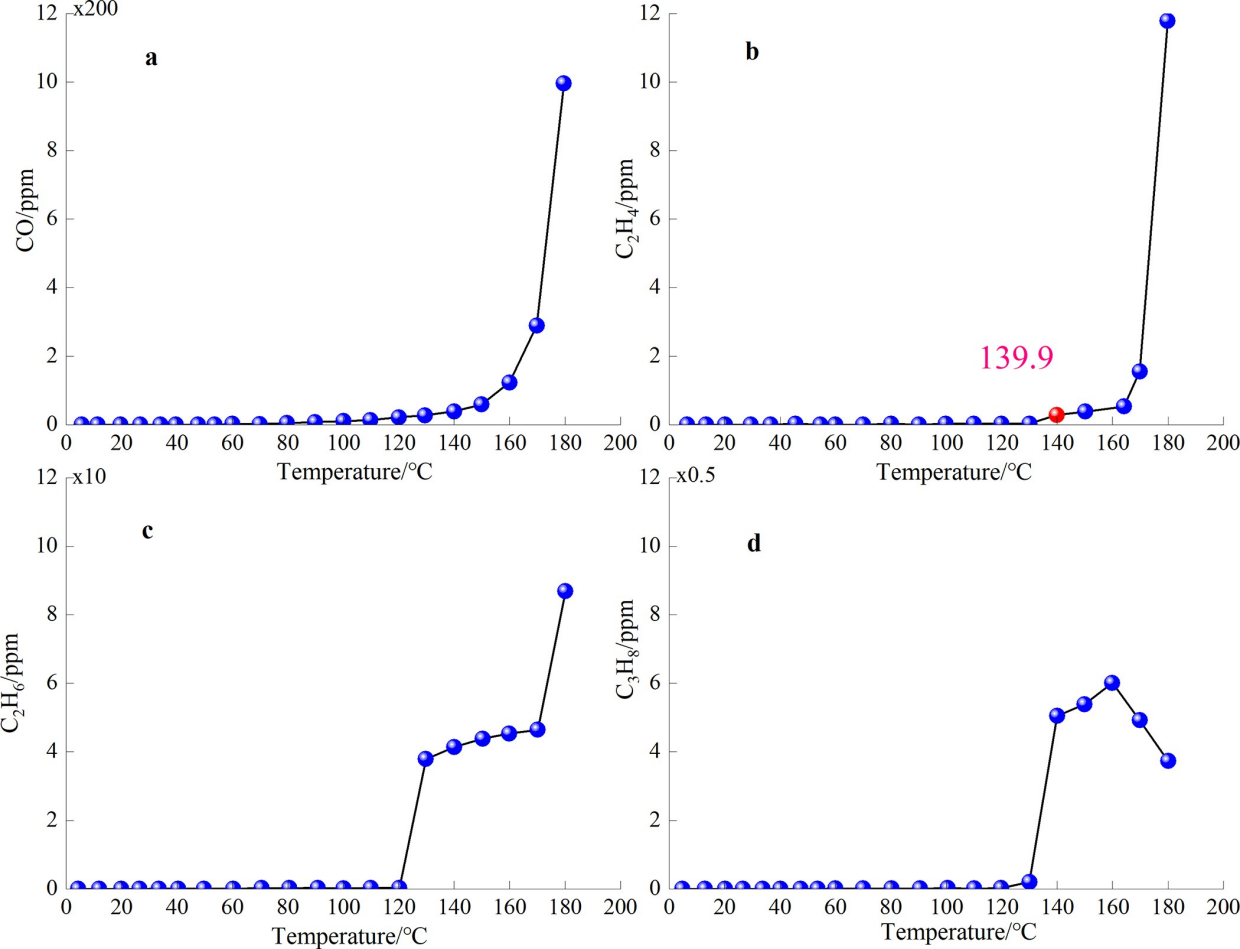
Variation of gas concentration from coal samples with temperature.

During the temperature programmed experiment, the critical temperature of CO, C_2_H_4_, C_2_H_6_, and C_3_H_8_ in coal samples were recorded, as illustrated in Fig 7.

Ethylene (C_2_H_4_) gas was detected at 139.9°C, and C_2_H_4_ gas was detected at 164.5°C, which then increased rapidly. Therefore, C_2_H_4_ (139.9°C) is regarded as the symbolic gas for coal spontaneous combustion to achieve severe oxidation and temperature rise.

### 3.3 Results of closed oxygen consumption test and analysis

**Fig 8:**
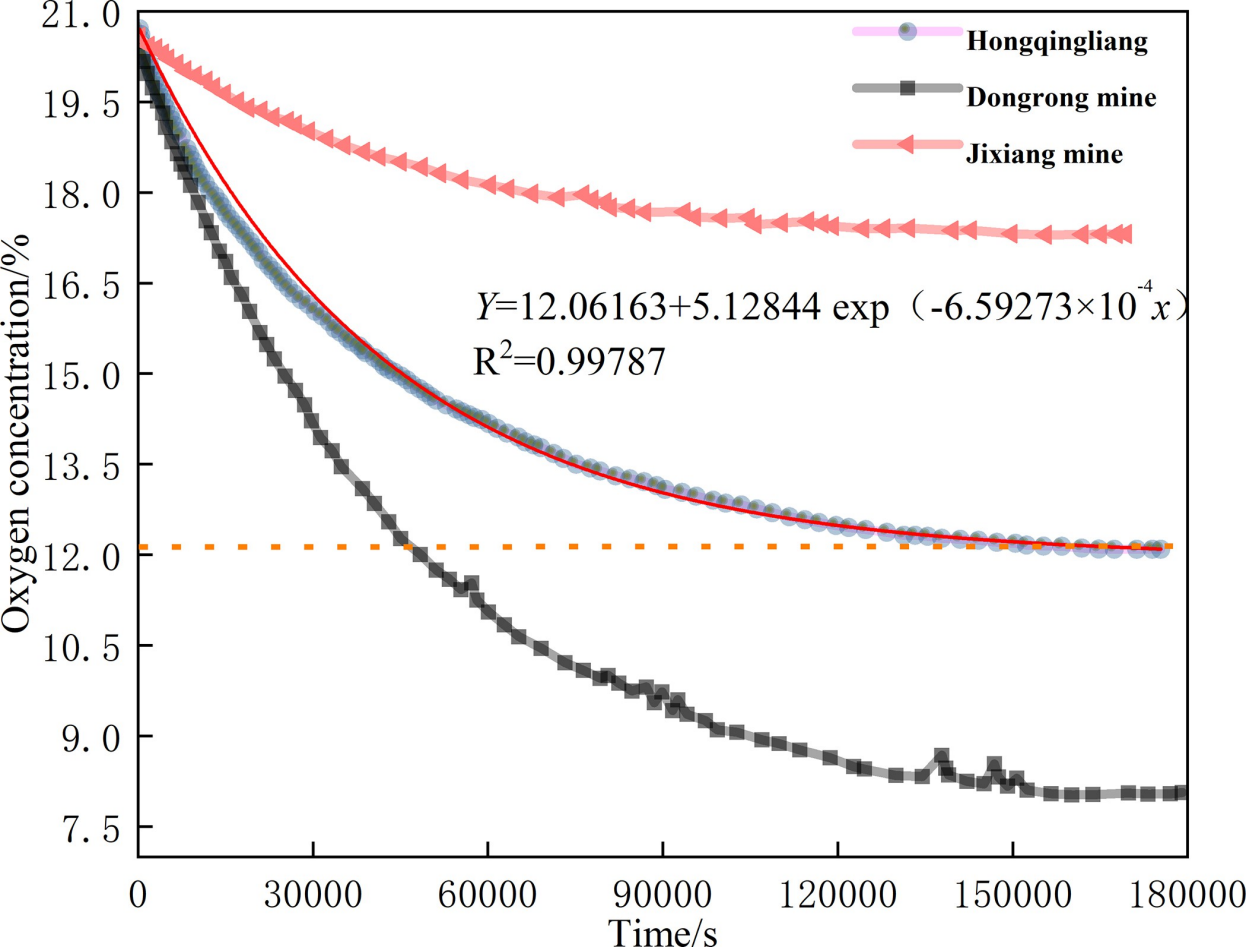
Experimental results of closed oxygen consumption of coal samples.

Fig 8 shows the closed oxygen consumption test results of coal samples from three mines. With the continuous reaction between coal and oxygen, the oxygen concentration in the experimental tank decreased continuously, showing a negative exponential change. The oxygen consumption capacity of coal samples differed in the three mines, the coal of Jixiang Mine and Dongrong Mine is bituminous coal, and the coal of Hongqingliang mine is lignite. When the oxygen concentration of coal samples in Dongrong Mine was approximately 12%, it stopped consuming oxygen and was finally stabilized at 12.06%. Hence, 12% is taken as the critical oxygen volume fraction of asphyxiation in the goaf of Dongrong Mine.

According to field experience, most scholars regard 10% oxygen concentration as the unified index for dividing the asphyxiation zone in a goaf [22–26]. Fig 8 shows that the closed oxygen consumption test results of coals in the different mines were different, indicating that the oxygen consumption capacity of different coals differs under the same conditions. Hence, it is unreasonable to take 10% as the critical oxygen volume fraction for all goafs.

A closed oxygen consumption test can not only measure the volume fraction of asphyxiated oxygen of coal samples and determine the boundary position of the asphyxiated zone in a goaf, but can also obtain the continuous oxygen consumption rate of coal samples [4], which can be used for subsequent goaf flow field simulations.

Assume that the oxygen concentration volume fraction *C(τ)* in the sealed tank approximately follows the distribution of the negative exponential function:

$$c(\tau )=c_{b}+(c_{0}‐c_{b})\cdot e^{-\lambda c\tau },$$ (3)

where *C*_*0*_ is the initial oxygen volume fraction, %; *λ*_*c*_ is the decay rate of the oxygen volume fraction, s^−1^; *C*_*b*_ is the stable oxygen volume fraction value, %; and *τ* is the oxidation time, s.

Derivation of time *τ*:

$$\gamma =\{\begin{array}{cc} 0 & ,(c(\tau )<c_{b})\\ ‐0.4464\lambda c(c_{0}‐c_{b})e^{-\lambda_{c}\tau } & ,(c(\tau )\geq c_{b}) \end{array},$$ (4)

where *γ* is the volumetric oxygen consumption rate, mol·(m^3^·s)^−1^; 0.4446 is the amount of substance per unit volume, mol·m^−3^.

**Fig 9:**
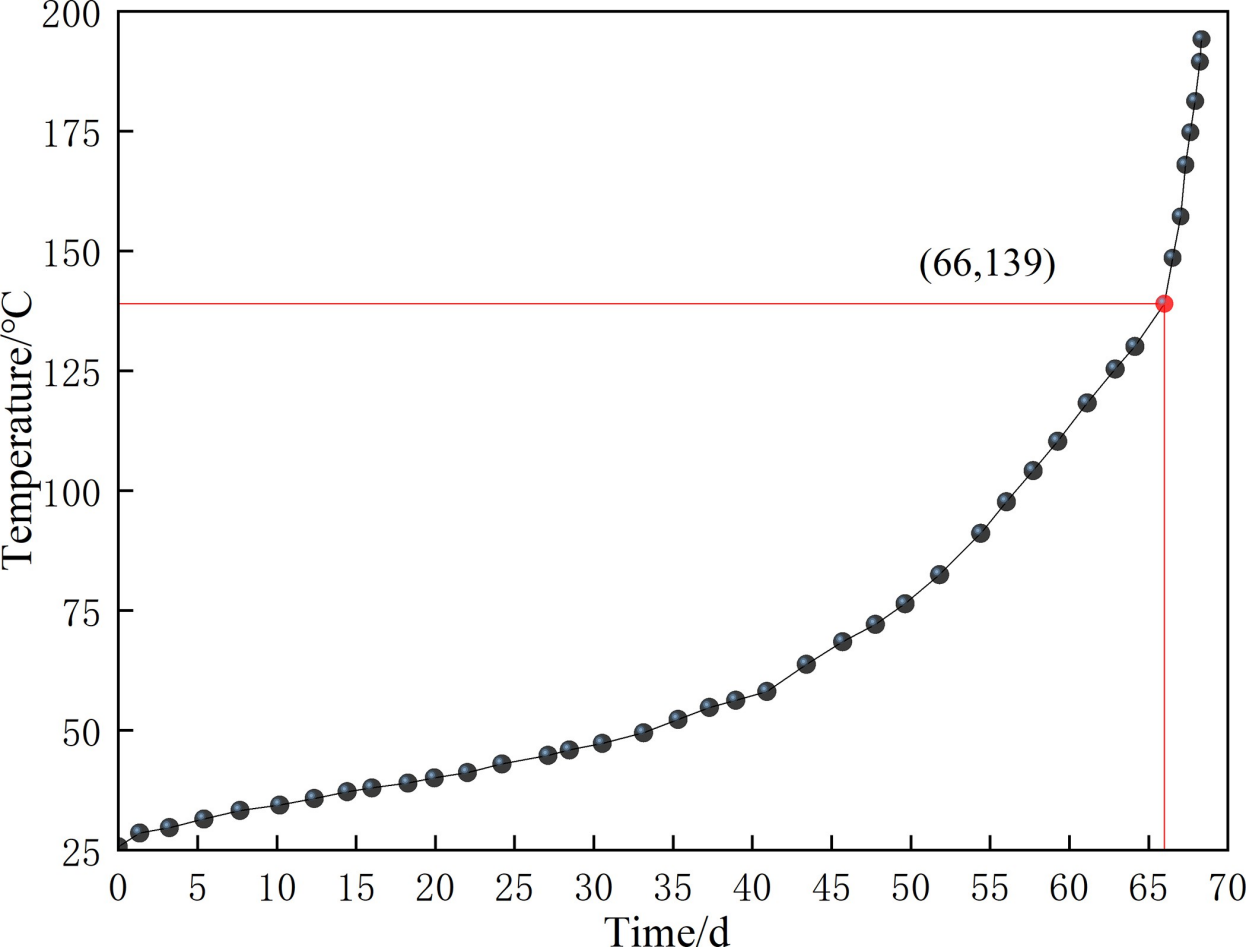
Variation of coal sample temperature with time.

Fig 9 illustrates the relationship between the temperature measured by the ventilation oxygen consumption test after the pipeline is disconnected and the change with time. The time corresponding to 139.9°C in the curve is the 66th day after the start of the coal sample heating experiment; thus, the spontaneous combustion ignition period of the coal sample is determined to be 66 days.

A control program (UDF) for oxygen consumption and temperature rise of residual coal in a goaf was developed for CFD simulation. The program was obtained by combining the simultaneous solution equation of multicomponent gas and temperature equation in reference [27], characteristic temperature of coal samples obtained from the coal sample oxidation experiment, fitting equation of kinetic parameters at the spontaneous combustion ignition stage, and the equation of the relationship between oxygen reduction and oxygen volume fraction in the suffocation zone (critical) of 12%.

### 3.4 Measured data and analysis

**Fig 10:**
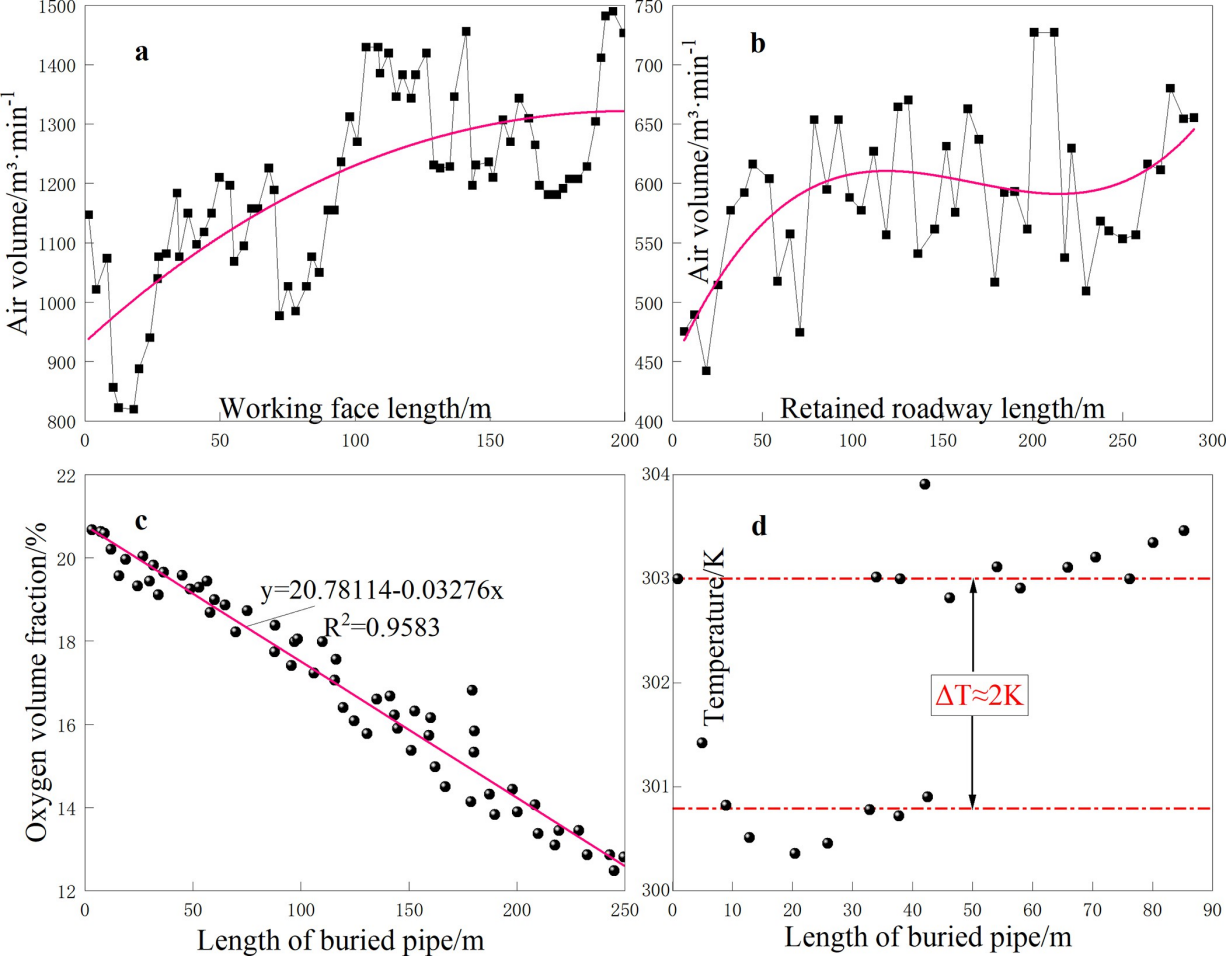
Measured field data.

Dongrong Mine is a low-CH_4_ mine. Fig 10 illustrates the air volume, temperature, and oxygen concentration measured in the goaf onsite.

Considering the coordinate axis marked in Fig 3, it can be concluded that the air volume from the air inlet port to the return air port of the working face gradually increased. The air leakage of the RCGSER retained roadway was mainly concentrated in the area approximately 100 m near the open-off cut and the working face.

Through a linear regression analysis, the relationship between the measured oxygen concentration value *C*_*i*_ and the position *L*_*i*_ from the working face in the goaf was obtained:

$$C_{i}=20.514-0.0267L_{i},$$ (5)

where *C*_*i*_ is the oxygen concentration value, %; *L*_*i*_ is the distance from the working face, m.

According to Eq 5, when the oxygen concentration on the return air side is 10%, the distance from the working face is *L*_i_ = 382 m, and the minimum fire prevention advancing speed of the working face should be greater than 5.8 meters per day when the spontaneous combustion ignition period is 66 days. As of the time of writing this paper, the advancing speed of the S2173 working face was 4 meters per day, and the spontaneous combustion prediction system did not monitor symbolic gases, such as ethylene and acetylene, indicating that spontaneous combustion did not occur in the goaf.

The temperature in the upper corner of the working face was approximately 29°C. The temperature in the deep area as well as the formation temperature tended to be stable, whereas the temperature in the shallow area was low. This phenomenon was due to the influence of ventilation in the working face, atmospheric temperature change, water evaporation, heat absorption, and ventilation cooling. The maximum deviation was approximately 2°C.

### 3.5 Software simulation results and analysis

#### (1) 3DEC simulation results of surrounding rock caving. Owing to space limitations, we only present the 3DEC simulation results of surrounding rock caving in the dip direction when the working face advanced 420 m, as shown in Fig 11

**Fig 11:**
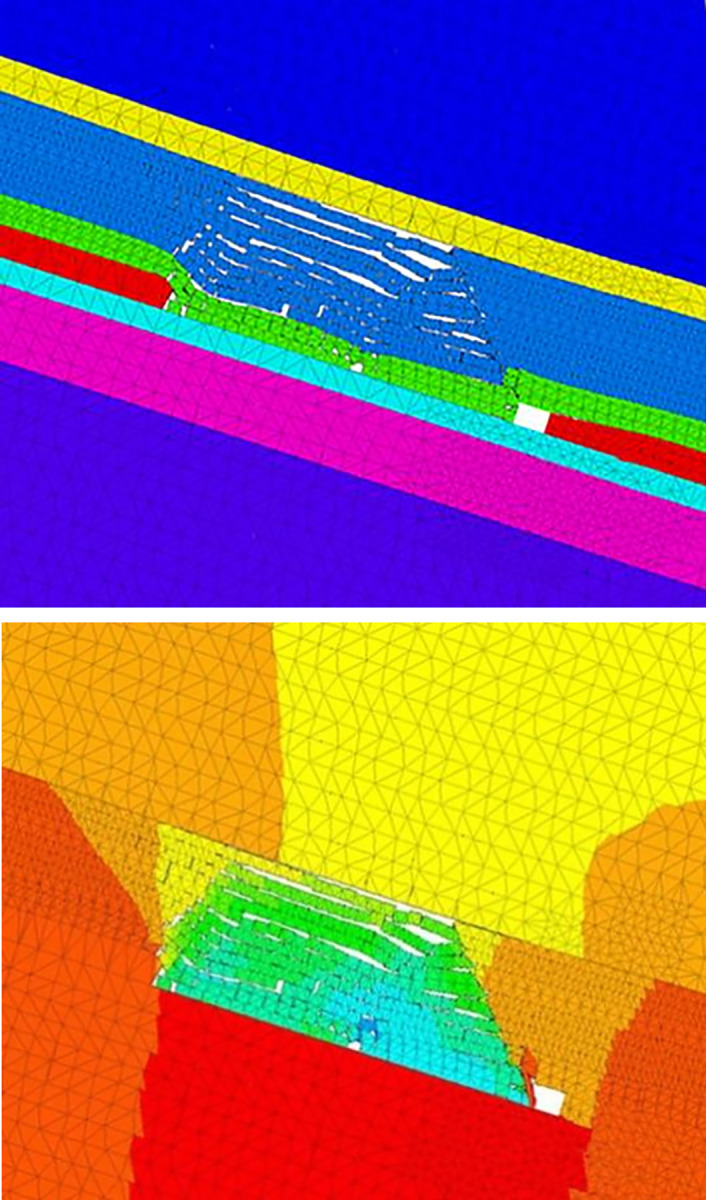
Simulation results of caving deformation of overburden in inclined direction.

**Fig 12:**
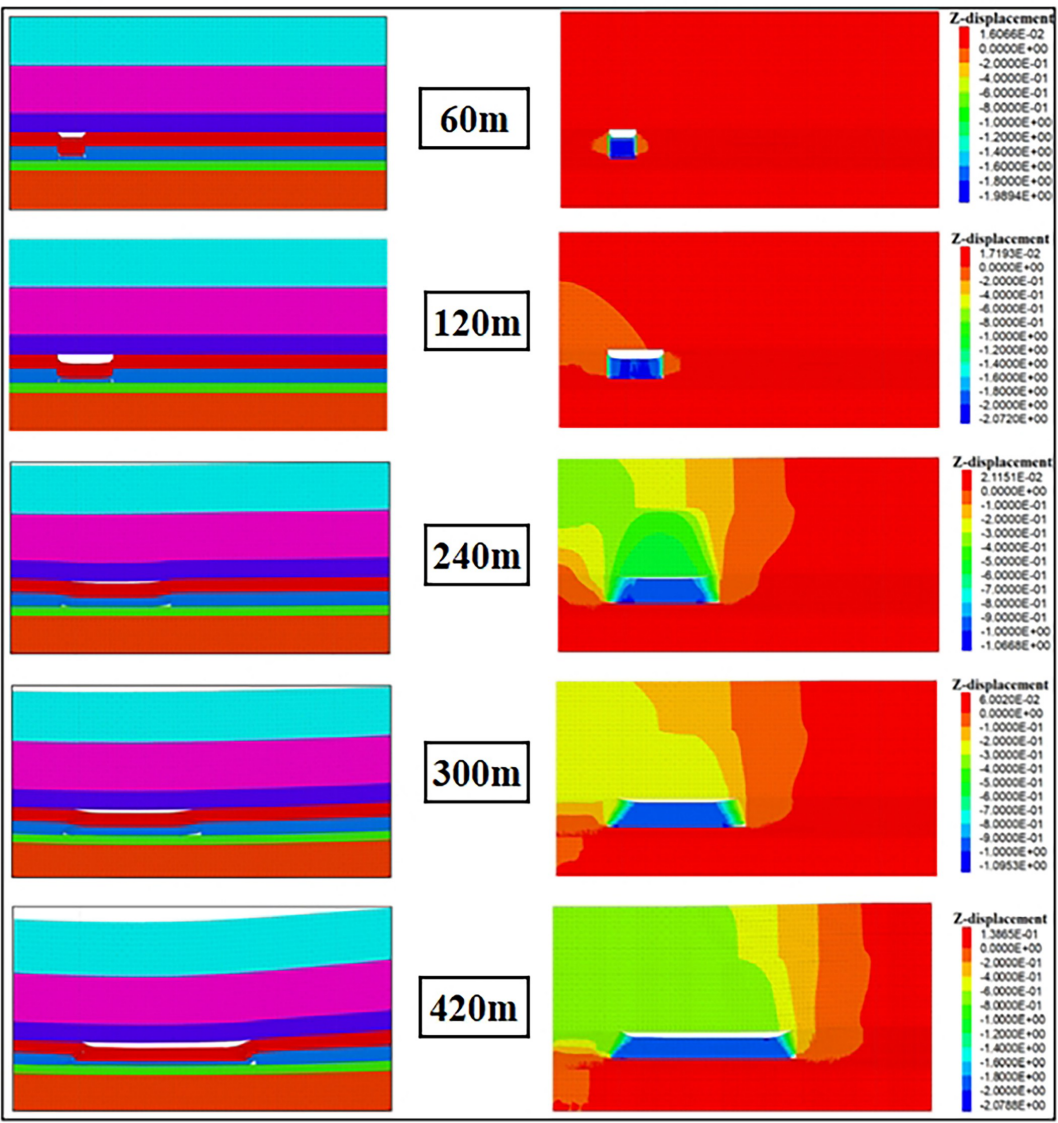
Simulation results of caving deformation of overburden in strike direction.

**Fig 13:**
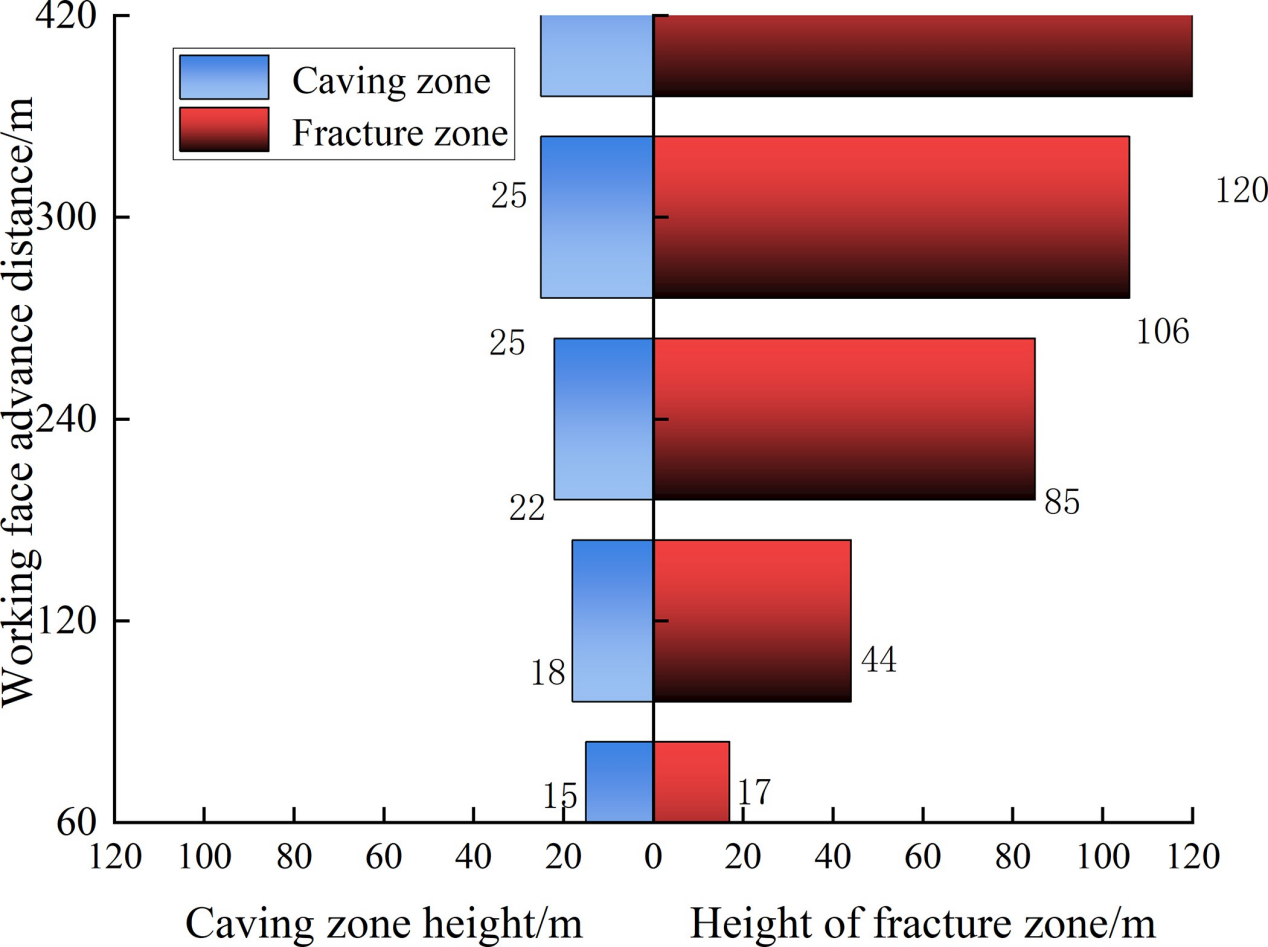
Relationship between overburden deformation and advancing distance of working face.

We also present the simulation results in the strike direction, as shown in Fig 12. In both figures, the right half is the vertical displacement diagram, the displacement is 0 in the initial state, and the corresponding color is red. According to the simulation results, Fig 13 illustrates the development height change map of the rock caving zone and fracture zone with increasing goaf strike length. Roof cutting improved the rock caving integrity on the retained roadway side. Compared with the return air side, the permeability and porosity near the retained roadway in the goaf were smaller. The height of the caving zone in the S2173 RCGSER working face was approximately 25 m after mining.

Considering the simulation results and previous research [28–30] and by adding a time parameter *t*, the three-dimensional time-varying porosity distribution characteristic equation the of RCGSER goaf was constructed. The surrounding rock caving control program (UDF) in CFD simulation was also compiled.

$$k_{p}=k_{p,min}+[k_{p,max1}+\varepsilon_{1}(k_{p,max2}-k_{p,max1})e^{-a_{2}(H-d_{2})}-k_{p,min}]e^{-a_{1}(d_{1}+tv+b_{1})(1-e^{-\varepsilon_{0}a_{0}(\alpha d_{0}+b_{0})})}\;d_{0}<\frac{L}{2}+1,$$ (6)

$$k_{p}=k_{p,min}+[k_{p,max1}+\varepsilon_{1}(k_{p,max2}-k_{p,max1})e^{-a_{2}(H-d_{2})}-k_{p,min}]e^{-a_{1}(d_{1}+tv+b_{1})(1-e^{-\varepsilon_{0}a_{0}(\beta L-\gamma d_{0}+b_{0})})}\;d_{0}\geq \frac{L}{2}+1,$$ (7)

$$n(k_{p},x,y,z)=1-1/k_{p},$$ (8)

where *n*(*k*_*p*_, *x*, *y*, *z*) is the porosity distribution; *k*_*p*_ is the coefficient of collapse and expansion of the mined-out area, dimensionless; *k*_*p*,*max*1_ is the initial caving breaking expansion coefficient; *k*_*p*,*max*2_ is the breaking expansion coefficient near the bottom plate at the working face; *k*_*p*,*min*_ is the coefficient of crushing expansion after compaction; *d*_0_ is the distance from a certain point to the filling wall on the side of the retaining roadway, m; *d*_1_ is the distance from a certain point to the working face, m; *d*_2_ is the distance from a certain point to the floor, m; *t* is time, s; *v* is the advancing speed of the working face, m/s; *H* is the height of the mined-out area, m; *L* is the working face length, m; *α*, *β*, *γ*, *ε*_0_, *ε*_1_, and *b*_1_ are adjustment factors, dimensionless; *a*_0_ and *a*_1_ are decay rates, dimensionless.

#### CFD simulation results

**Fig 14:**
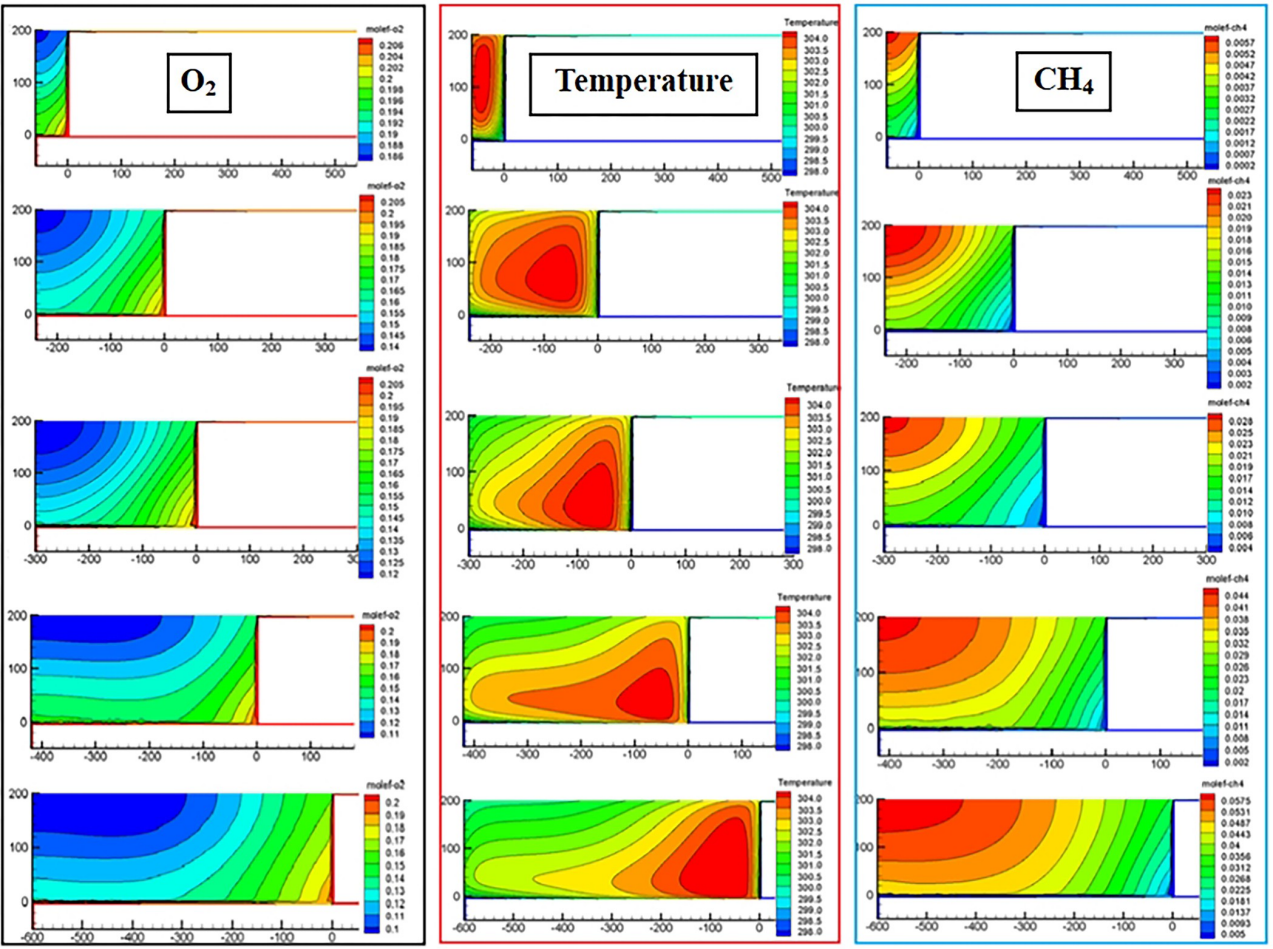
CFD simulation results of ambient temperature 298 K.

**Fig 15:**
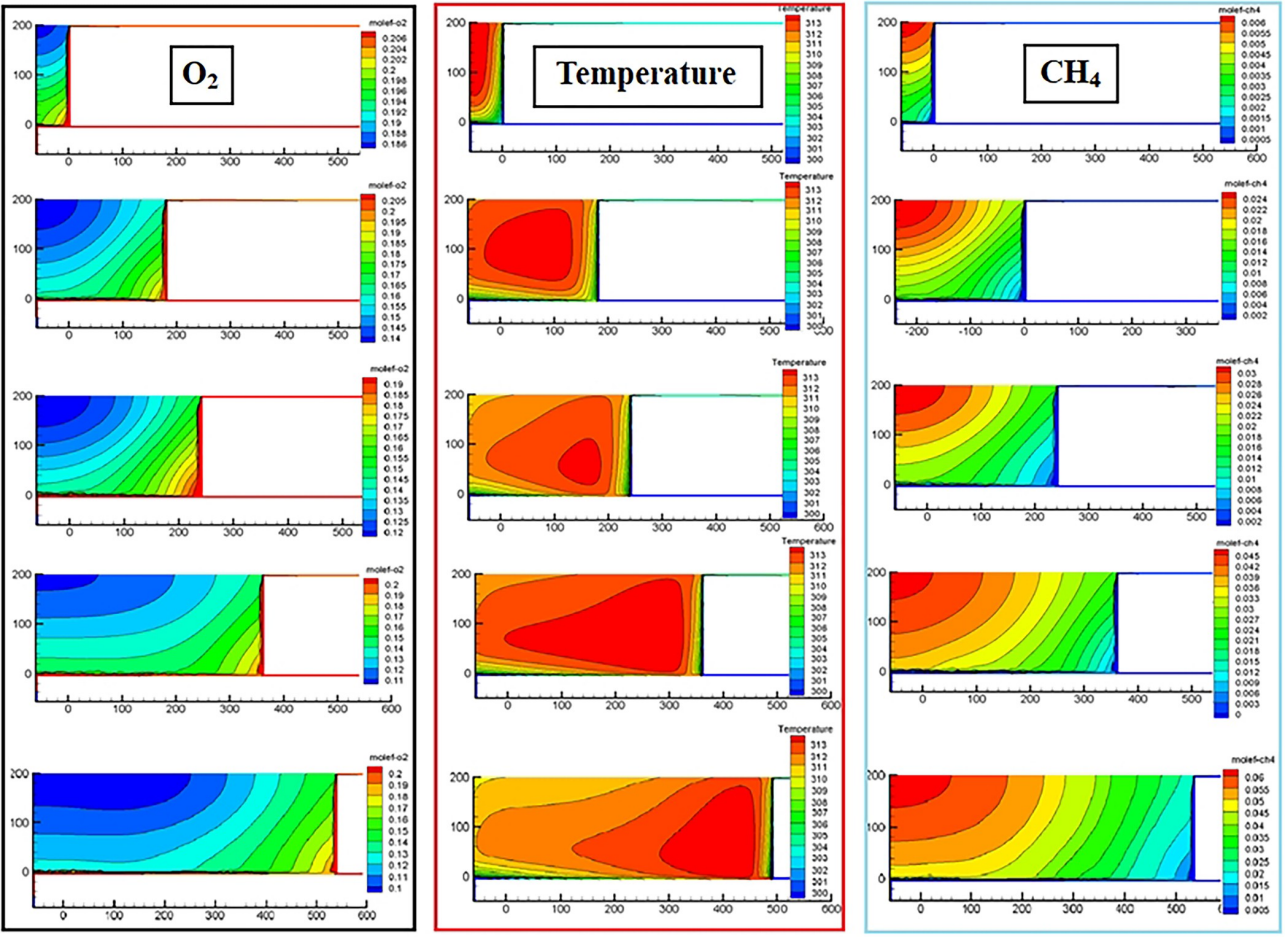
CFD simulation results of ambient temperature 305 K.

The distribution of the oxygen field and temperature field in the Z = 0.5 m slice of the goaf with different ambient temperatures (different mining levels) simulated by CFD is shown in Figs 14 and 15. The distribution result of the CH_4_ field was selected from the Z = 18.5 m slice. Owing to space limitations, we present only the simulation results of the working face advancing to 60 m, 240 m, 300 m, 420 m, and 600 m.

According to the results presented in Fig 8, the sites with an oxygen volume fraction greater than 12% in the goaf were classified as SCD areas. Figs 14 and 15 show that the distribution form and range of oxygen, temperature, and gas fields in the RCGSER goaf changed constantly with increasing goaf strike length. The high-temperature area gradually approached the lower corner of the working face, gradually presenting a strip distribution close to the retaining roadway. With the increase in mining depth (environmental temperature), the distribution range of the SCD area in the goaf was reduced, but the extreme temperature value and distribution range in the high-temperature area increased.

**Fig 16:**
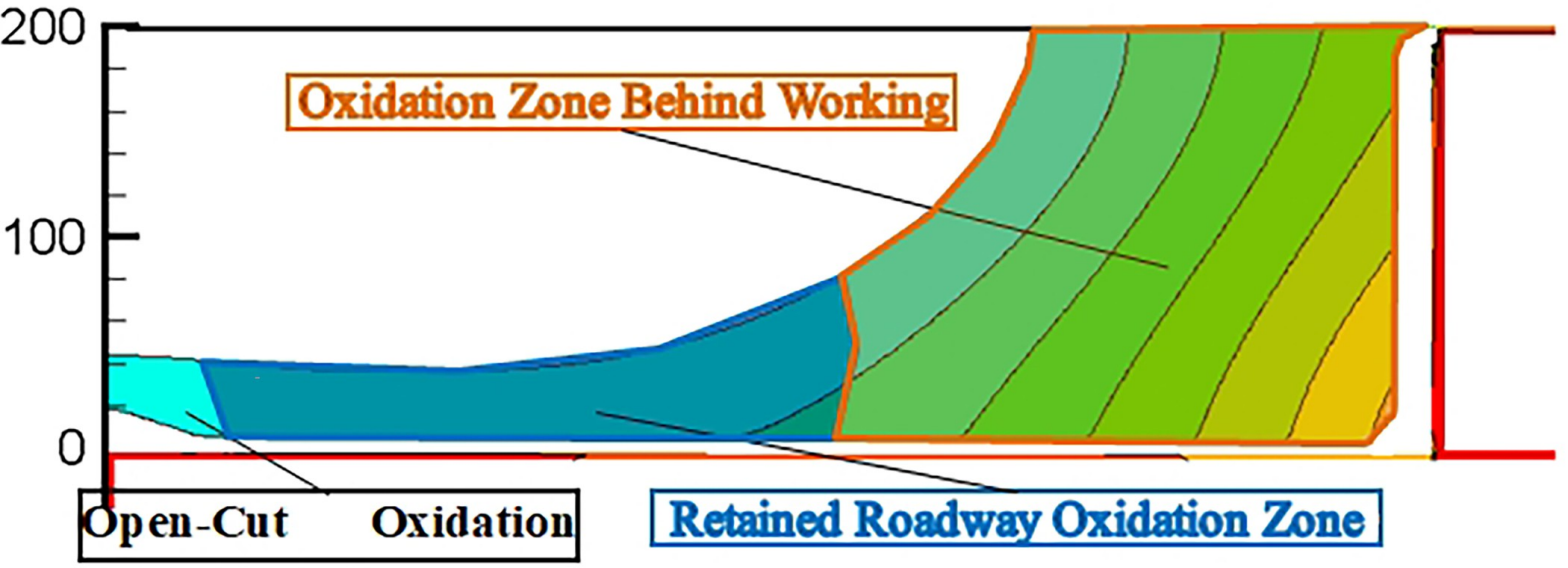
Division of spontaneous combustion oxidation zone in RCGSER goaf.

Based on the simulation results, the following conclusions can be drawn:

1. The RCGSER first working face goaf exhibits three distinct potential spontaneous combustion disaster (SCD) areas: (i) the region adjacent to the open-off cut, (ii) the area within 20 to 60 meters near the retained roadway, and (iii) the zone behind the working face. The distribution pattern and extent of these areas continuously evolve as the strike length of the goaf increases. Fig 16 illustrates these three zones, which are referred to as the "Open-Cut Oxidation Zone," the "Retained Roadway Oxidation Zone," and the "Oxidation Zone Behind Working Face."
2. The pressure difference caused by air leakage in the working face affects the flow of air into the RCGSER goaf, and the action range is the retained roadway section 0––100 m close to the working face.
3. The oxygen volume fraction was always high within 20–60 m near the retained roadway, and the width of this area extended continuously with increasing goaf strike length. Hence, the “Retained Roadway Oxidation Zone” presents a considerable threat to the RCGSER goaf.

**Fig 17:**
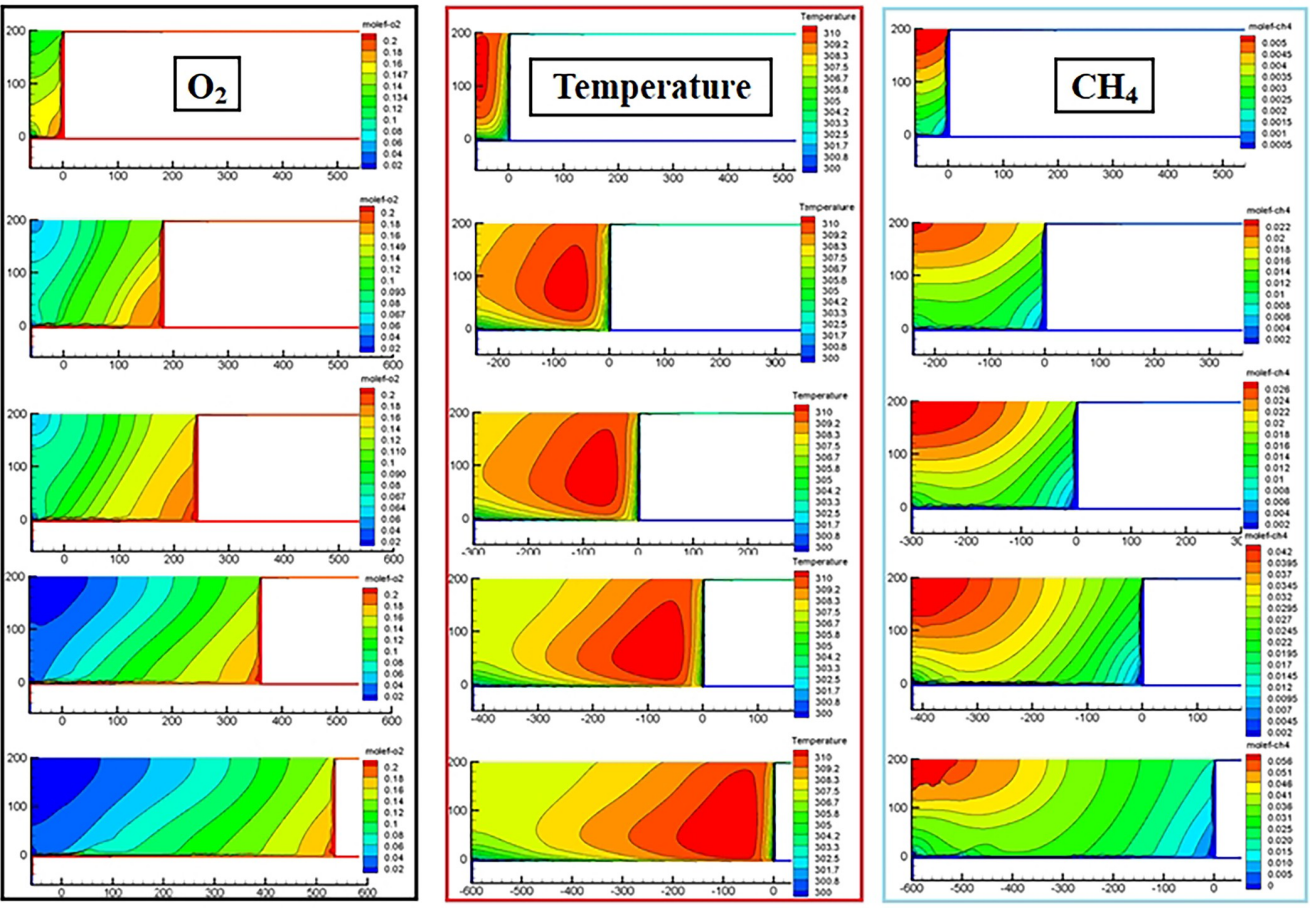
Simulation results after nitrogen injection.

The CFD simulation of nitrogen injection was performed on the basis of an ambient temperature of 305 K; the simulation results are illustrated in Fig 17.

**Fig 18:**
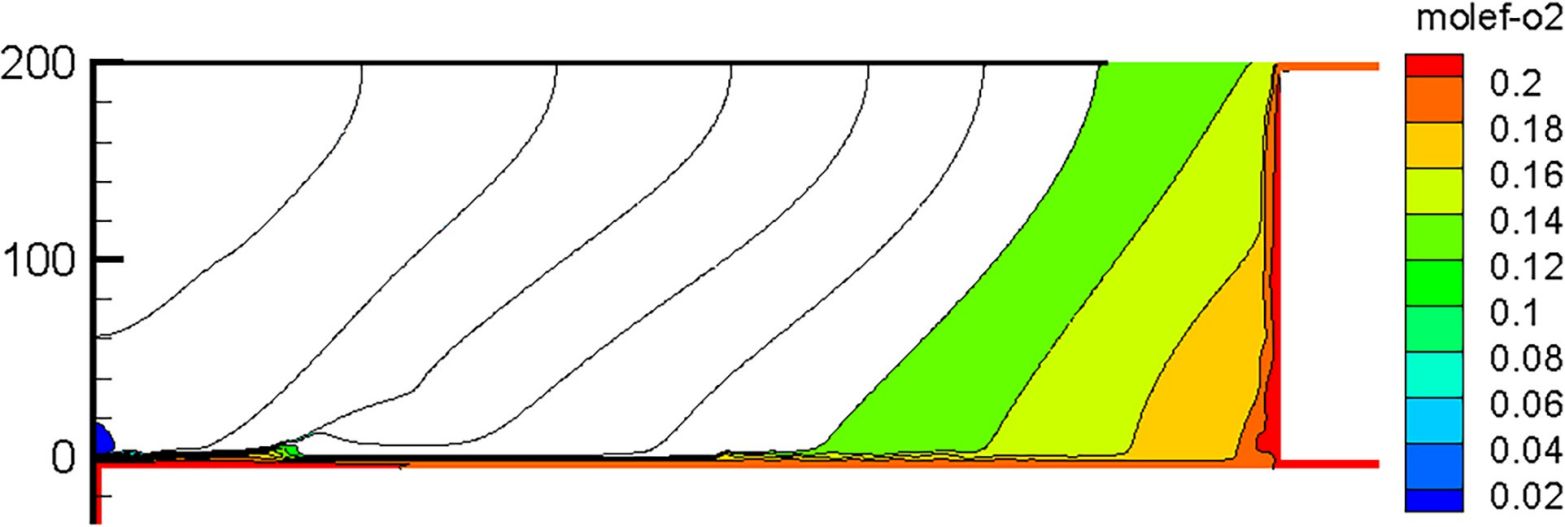
Distribution of oxygen flow field in goaf after nitrogen injection.

After nitrogen injection, the distribution patterns and ranges of oxygen, gas, and temperature fields in the goaf changed. Fig 18 illustrates the simulation results of the working face advancing 600 m after nitrogen injection. After nitrogen injection in the tail roadway, the Open-Cut Oxidation Zone and Retained Roadway Oxidation Zone were eliminated, and the distribution range of the Oxidation Zone Behind Working Face was reduced, indicating that the measure of nitrogen injection in the tail roadway was effective.

**Fig 19:**
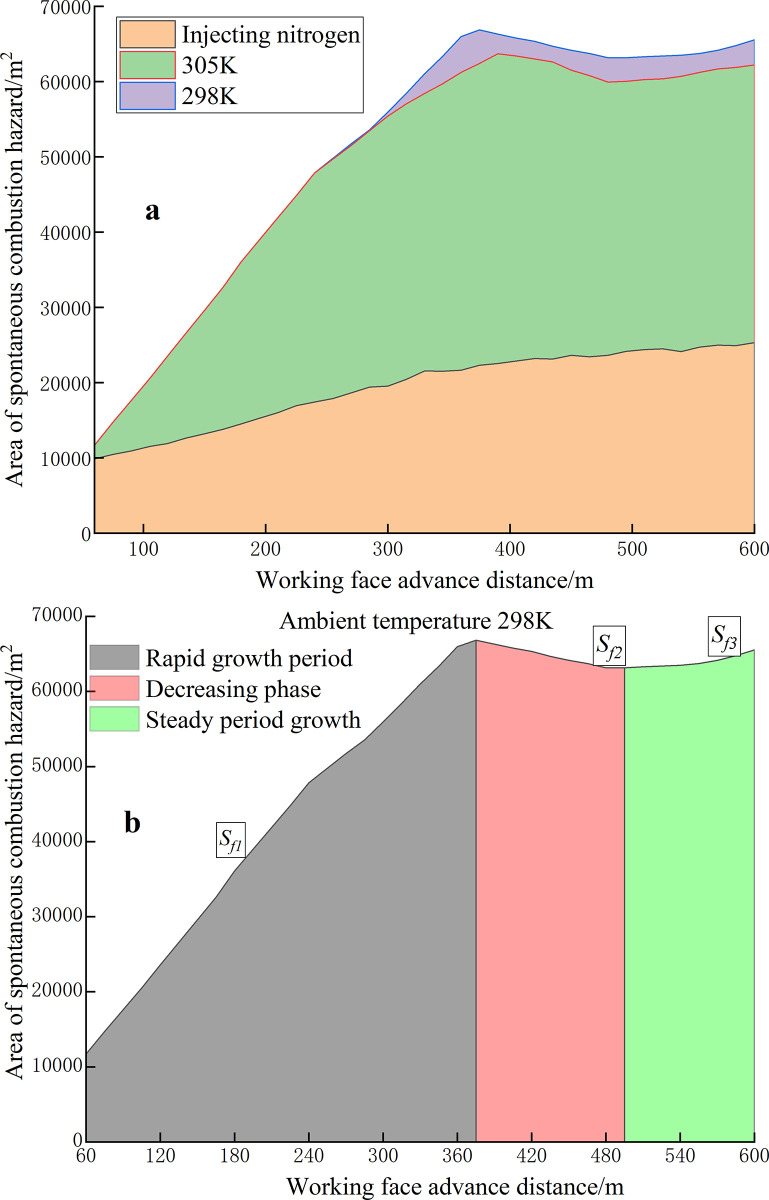
Area variation curve of SCD range.

To analyze the dynamic distribution of spontaneous combustion in the RCGSER goaf more intuitively and quantitatively, the Z-axis of the SCD area was projected in the CFD simulation results. As shown in Fig 19, the value of the SCD area varied with the advancing of the working face.

By fitting the 298k area curve, the following equations are obtained:

$$S_{f1}=-680275.9+112005.6\ln(x+418.8)(R^{2}=0.99),\;(60<x<375)$$ (9)

$$S_{f2}=20617.0•e^{-x/232.5}+27977.6•e^{-x/232.5}+57213.0(R^{2}=0.98),\;(375\leq x\leq 495)$$ (10)

$$S_{f3}=1.63\times 10^{-4}•e^{x/36.2}+63040.5(R^{2}=0.99).\;(495<x<600)$$ (11)

where *S*_*f*_ is the area of the spontaneous combustion hazard, m^2^; *x* is the distance from the working face, m.

“Double inflection points” are observed in the area change curves of the SCD area at an ambient temperature of 298 K and 305 K: the change in the SCD area in the RCGSER goaf undergoes three stages: rapid growth period, decline period, and stable growth period. In the stable growth period, although the area of the dangerous zone increases, the point of increase is 20–60 m near the retained roadway, and the range increases with increasing goaf strike length. After nitrogen injection, the area of the SCD zone increases slightly, and then it gradually tends to be stable.

## 4. Conclusions

1. The results of the coal sample oxidation experiment showed the following: the water loss endpoint temperature of the Dongrong coal sample was 170°C, the ignition temperature was 320°C, the fitting equation of kinetic parameters in the spontaneous combustion ignition stage is Y=16.97792−16929.99674X,, the equation of oxygen concentration attenuation is $Y=15.06163+5.12844\;exp(-6.59273\times 10^{-4}x)$, the shortest spontaneous combustion ignition period is 66 days, and the critical oxygen volume fraction is 12%. Using the above results, we compiled the control program of oxygen consumption and temperature rise of residual coal in CFD simulation.
2. Using the 3DEC software, the evolution law of overlying rock fractures in the goaf of S2173 first mining face in Dongrong Mine was obtained. The stable height of the caving zone was 25 m, and the caving overlying rock on the side of the retaining roadway was strongly integrated by roof cutting; the permeability and porosity in this area were small. According to the simulation results, a four-dimensional mathematical model of porosity and permeability of porous media in the goaf was constructed.
3. As the working face advanced, the distribution range of the SCD area in the RCGSER U + L ventilated goaf increased first, decreased afterward, and finally increased steadily; the corresponding area curve had “double inflection point.” Three spontaneous combustion oxidation zones were in the goaf: “Open-Cut Oxidation Zone,” “Retained Roadway Oxidation Zone,” and “Oxidation Zone Behind Working Face.” The “Retained Roadway Oxidation Zone” had considerable influence on the gob spontaneous combustion, which was eliminated by the proposed measure of injecting nitrogen in the tail roadway. Thus, spontaneous combustion in the goaf can be prevented to ensure mine safety during production.
